# Dr. Eugene Grissom

**Published:** 1902-08

**Authors:** 


Dr. Eugene Grissom, the distinguished alienist formerly of North Carolina, in a state of temporary mental aberration, took his own life at his son's residence, Washington, D.C., July 27, 1902, aged 71 years. He served for twenty-one years as superintendent of the North Carolina Insane Hospital and achieved a national fame in the study and management of mental diseases, only to succumb to its results.

